# Laparoscopic central pancreatectomy for solid pseudopapillary tumors of the pancreas: our experience with ten cases

**DOI:** 10.1186/1477-7819-12-312

**Published:** 2014-10-13

**Authors:** Xue-Min Chen, Yue Zhang, Dong-Lin Sun

**Affiliations:** Department of Hepatobiliary Surgery, The Third Affiliated Hospital of Soochow University, Changzhou, 213003 China

**Keywords:** Solid pseudopapillary tumor, Pancreatic tumor, Laparoscopic central pancreatectomy

## Abstract

**Background:**

Solid pseudopapillary tumors (SPTs) of the pancreas are a rare neoplasm. There are few reports of laparoscopic central pancreatectomies (LCPs) for SPT of the pancreas. The objective of this study was to evaluate the feasibility, safety and long-term outcome of LCP based on a series of SPT patients.

**Methods:**

This retrospective study included ten patients who underwent LCP between 2009 and 2013. Clinical characteristics and intra- and postoperative data were retrospectively analyzed. A follow-up of at least 3 months was available for all patients.

**Results:**

All procedures were successfully performed laparoscopically, and no patient required intraoperative blood transfusion. The median operative time was 271 min (range 250 to 310 min) and the median loss of blood was 104 ml (range 80 to 150 ml). The mean tumor size was 51 mm (range 38 to 62 mm). All patients underwent complete resection with negative surgical margin. An average of 5.8 lymph nodes were resected without metastases. The median first flatus time was 2 days, and the median starting time for diet was 3 days. The median postoperative hospital stay was 13 days (range 10 to 23 days). Morbidity was 20%. The median follow-up was 22.9 months (range 3 to 48 months), at which point all patients were alive with no recurrence. None of the patients developed exocrine or endocrine insufficiency. No hospital mortalities occurred in our patient group.

**Conclusions:**

LCP is a safe and effective technique for resecting SPT of the neck and proximal body of the pancreas while preserving pancreatic endocrine and exocrine function, and the spleen.

## Background

Solid pseudopapillary tumors (SPTs) are a rare clinical entity, representing 1% to 2% of all primary exocrine tumors of the pancreas; more than 80% of patients are female [1]. SPT is of unclear histopathogenesis, and low-grade malignancy, malignant degeneration and lymph node metastasis rarely occur [2]. Surgical resection of this tumor can result in long-term survival. Laparoscopic resection of the pancreas was initially described in the medical literature in the early 1990s. The first laparoscopic pancreatoduodenectomy was performed in 1994, and the first distal pancreatectomy was performed in 1996 [3, 4]. However, patients who require central pancreatectomy are still being treated with the open approach or with laparoscopic distal pancreatectomy. Central pancreatectomy is an alternative technique for benign or low-grade malignant tumors of the neck of the pancreas. This pancreas-sparing technique was developed to avoid exocrine and/or endocrine insufficiency, which could be detrimental to the patient’s quality of life, especially for benign or low-grade malignant neoplasms.

Although laparoscopic central pancreatectomy (LCP) is thought to be a function-preserving and minimally invasive pancreatectomy, due to the difficulty of pancreaticoenteric reconstruction, LCP has been slow to gain popularity. In an attempt to define the role and the efficacy of minimally invasive surgery in the treatment of SPT of the pancreas better, we therefore describe a complete LCP.

## Methods

We undertook a retrospective cohort study of patients treated for SPT in our institution between February 2009 and December 2013 (*n* =15). Of the patients, 66.6% (10/15) were treated using LCP, and the other five patients underwent a laparoscopic distal pancreatectomy. Descriptive data were collected. Preoperative variables include age, gender and indication for surgery. Pancreatic fistula, delayed gastric emptying and post-pancreatectomy hemorrhage were defined according to the International Study Group of Pancreatic Surgery definitions [5–7]. Data collected included patient characteristics, operative details, morbidity and mortality, postoperative hospital stay, pathological findings and follow-up results. Oncologic outcomes were analyzed for all patients, and the data includes tumor size (maximum dimension in centimeters), total number of lymph nodes, number of positive lymph nodes and margin status. The fasting blood glucose level (normal ≤110 mg/dL) was used to evaluate the pancreatic endocrine function. A clinical evaluation was used to assess the pancreatic exocrine function. Patients with diarrhea, weight loss and fatty stools were considered to have pancreatic exocrine insufficiency. Ethics committee of the third affiliated hospital of Soochow University approval and informed consent from the patients were obtained to perform LCP.

### Operative technique

**Figure 1:**
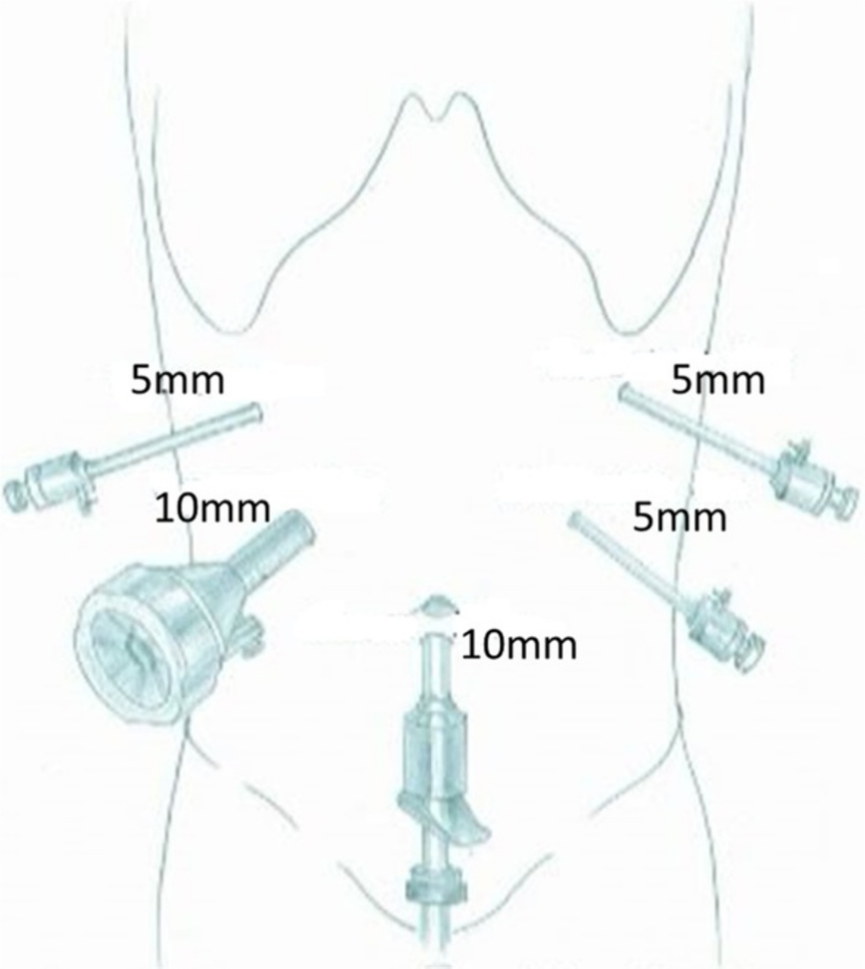
**Placement of trocars.**

**Figure 2:**
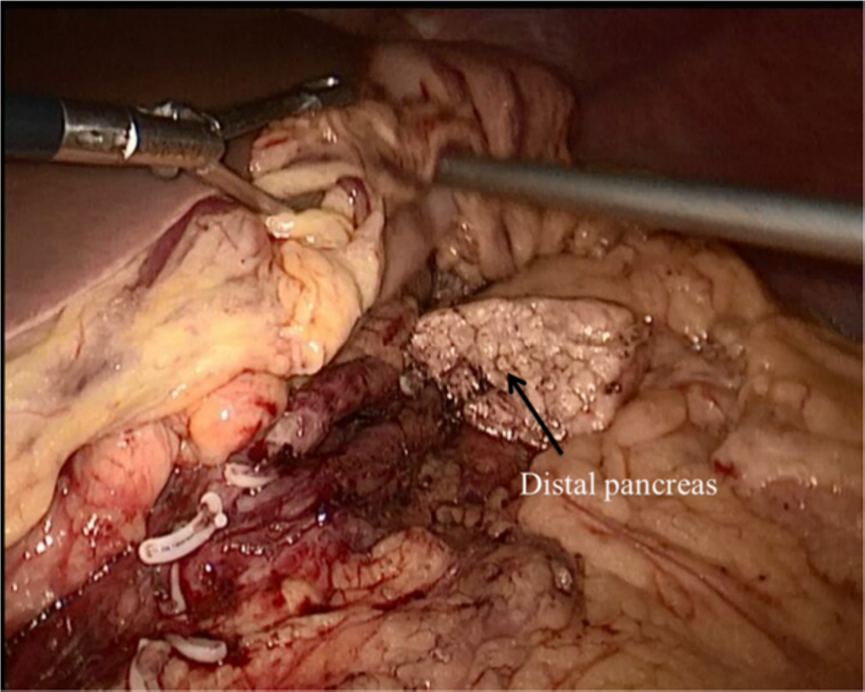
**Pancreas dissection and transection performed by laparoscopic central pancreatectomy.**

The LCP was performed with the patient in the supine and 30° anti-Trendelenburg position with the surgeon standing between the patient’s legs. Five trocars (three 5-mm and two 10-mm trocars) were inserted into the upper abdominal quadrant. We used a supra-umbilical cutdown to establish pneumoperitoneum, with a 5-mm port and a 10-mm port in the left upper and left flank quadrants, and two 5-mm ports in the right upper and right flank quadrants (Figure 1). We find such port placements are ergonomically good and allow adequate exposure. Under pneumoperitoneum, the gastrocolic ligament was divided to give access to the lesser sac using a Harmonic scalpel (Harmonic Ace; Ethicon Endo-Surgery, Cincinnati, OH, USA). The inferior border of the pancreas was dissected carefully using a blunt instrument to create a tunnel and expose the portal vein. The superior border of the pancreas was then dissected, and a tape was passed around the neck of the pancreas. The next step was to dissect the proximal and distal parts of the neck of the pancreas. This can be achieved by removing the lymph nodes along the hepatic, the gastroduodenal and the splenic arteries. At this point, the splenic vessels and portal vein were completely dissected free from the pancreatic neck. The pancreas was transected with an endoscopic linear stapler (Endocutter 60 stapler, white or blue cartridge; Ethicon Endo-Surgery, Cincinnati, OH, USA) on the right side of the tumor followed by transection of the distal pancreas (Figure 2). The LCP was now complete. The surgical specimen was put inside a plastic bag and removed through the umbilical port. The specimen was examined, and frozen sections were used to evaluate the surgical margins.

**Figure 3:**
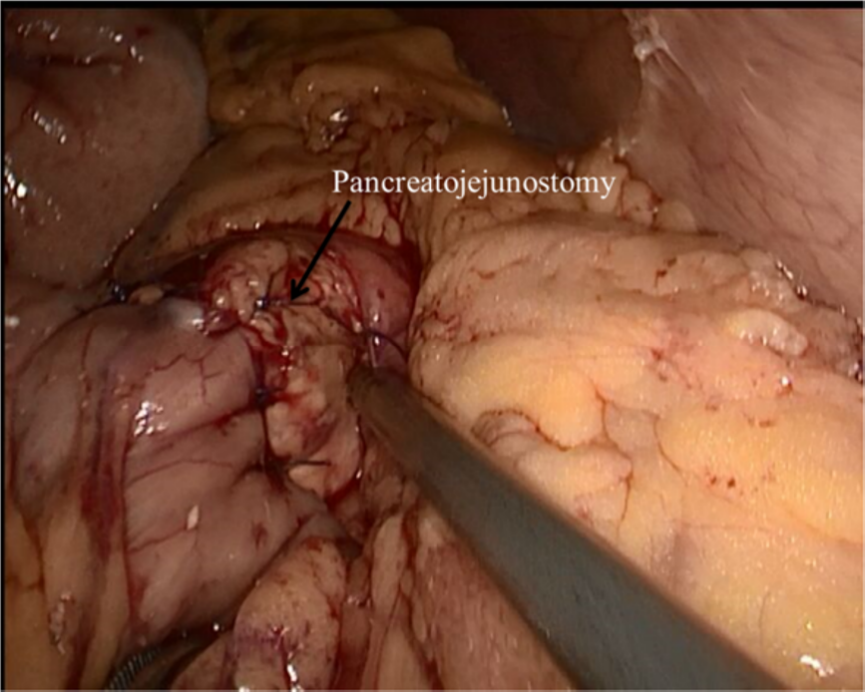
**Pancreaticojejunostomy.**

After completing the pancreatic resection, a Roux-en-Y jejunal loop was prepared. The jejunum was identified and divided using a stapler, 40 cm from the Treitz ligament. An end-to-side pancreatojejunostomy was then performed. A row of 3-0 coated polyglactin 910 sutures (Vicryl^™^; Ethicon Products, Johnson & Johnson, Somerville, NJ, USA) with interrupted stitches was placed between the jejunal serosa and the posterior side of the pancreatic capsule for apposition. The jejunum was opened with a Harmonic scalpel, suitable for a distal pancreatic stump. The posterior layer was performed with a continuous or interrupted 3-0 Vicryl suture between the pancreas (parenchyma and capsule) and the full thickness of the jejunum. The anterior layer was performed in the same way as the posterior layer (Figure 3). Finally, a side-to-side jejunojejunostomy was performed with an endoscopic linear stapler (Endocutter 60 stapler, white cartridge). The abdominal cavity was reviewed and drained.

## Results

LCP was performed successfully for all the patients. The splenic vessels and the spleen were preserved in all patients. Perioperative data are shown in Table 1. Their median age was 44.6 years (range 35 to 57 years). Eight patients were female and two male. One patient was admitted because of epigastric pain for 1 week; the others were found by chance during routine physical exams, and were asymptomatic. The median operative time was 271 min (range 250 to 310 min) and the median loss of blood was 104 ml (range 80 to 150 ml). The mean tumor size was 51 mm (range 38 to 62 mm). All patients received complete resection with a negative surgical margin. An average number of 5.8 lymph nodes were resected without metastases. The median first flatus time was 2 days, and median starting time for diet was 3 days. The median postoperative hospital stay was 13 days (range 10 to 23 days). Two patients experienced a postoperative pancreatic fistula (grade A), which was managed conservatively and ultimately cured, and the drainage tubes were removed on postoperative days 17 and 20, respectively. The hospital stay for these patients was 20 days and 23 days, respectively. The other patients recovered well after surgery. The median follow-up was 22.9 (3 to 48) months at which point all patients were alive with no recurrence. None of the patients developed exocrine or endocrine insufficiency. No hospital mortalities occurred in our patient group.

**Table 1:** **Surgery-related information and postoperative outcomes**

Case	Age/gender	Operative time (min)	Blood loss (ml)	Postoperative complication	Hospital stay (days)	Tumor size (mm)	Lymph nodes	Follow-up (months)	Exocrine or endocrine insufficiency
1	42/F	250	100	–	10	48	5	28	None
2	35/F	280	110	–	10	51	6	36	None
3	43/F	240	90	–	11	50	5	48	None
4	40/F	300	120	Pancreatic fistula	20	60	7	12	None
5	52/F	270	90	–	10	54	6	30	None
6	57/F	260	100	–	12	55	5	30	None
7	44/F	260	90	–	11	50	6	24	None
8	45/M	290	110	–	12	38	5	6	None
9	40/F	250	80	–	12	42	6	3	None
10	48/M	310	150	Pancreatic fistula	23	62	7	12	None

F, female; M, male.

## Discussion

Laparoscopic pancreatic surgery has undergone significant development in the last few years. The majority of procedures are left pancreatectomy and enucleations [8–10]. More complex pancreatic resections such as pancreatoduodenectomies, resections of the uncinate process of the pancreas and central pancreatectomies are performed routinely in very few centers [11, 12].

The first surgical resection of a pancreatic SPT was performed in 1970 and the first laparoscopic SPT resection was in 2003 [13, 14]. The first series of laparoscopic SPT resection (ten cases) was published by Cavallini *et al*. [15] in 2011. However, there have been few reports of LCP for SPT of the pancreas. Most of these articles are case reports and small series. The two largest series of LCP, with six and nine cases, respectively, reported morbidity rates of 33.3% and 33.3% with pancreatic fistula rates of 33.3% and 22.2%, respectively, with no mortality [16, 17]. A review of 512 patients from 21 series who underwent an open central pancreatectomy reported an overall morbidity rate of 41% (range 13% to 62%), a pancreatic fistula rate of 27% (range 0% to 62%) and a reoperation rate of 4% (range 0% to 21%) [18]. Our series with ten cases had a morbidity rate of 20% (two cases of a pancreatic fistula), similar to what has been reported. In a comparative study, the outcomes after a central pancreatectomy were compared with a control group that underwent extended left pancreatectomy for neoplasms in the mid pancreas [19]. After a median follow-up of 54 months, the incidences of endocrine and exocrine insufficiency after the central pancreatectomy were 4% and 5%, respectively, compared to 38% and 15.6% in patients who underwent an extended distal pancreatectomy. In this study, we have not observed any recurrence or pancreatic endocrine or exocrine insufficiency.

Laparoscopic resection of the neck of the pancreas or of any segment in the middle of the pancreas is not difficult. However, it entails reconstruction of the main pancreatic duct, which may be difficult and sometimes hazardous laparoscopically. The popularity of laparoscopic left pancreatectomy has certainly reduced the number of patients undergoing LCP. However, this is at the expense of the endocrine and exocrine deficiency that an extended left pancreatectomy may produce. For benign or low-grade neoplasms, a left pancreatectomy may remove too much of the functioning pancreatic parenchyma. Due to this, for cases with a tumor in the neck of the pancreas, our procedure of choice is a central pancreatectomy with Roux-en-Y pancreatojejunostomy. The management for a distal pancreas can be pancreatogastrostomy or Roux-en-Y pancreatojejunostomy. Pancreatogastrostomy is easier and faster, but it may delay oral feeding and it prolongs the length of stay. Pancreatojejunostomy is a more complex reconstruction, but has better long-term outcomes in terms of endocrine and exocrine function. As central pancreatectomy is indicated in patients with an expected long survival, some authors consider pancreatojejunostomy as the best management for the distal pancreas after central pancreatectomy. We also prefer reconstruction with Roux-en-Y pancreatojejunostomy.

Depending on whether the duct of Wirsung could be identified, we used two methods to accomplish the pancreaticojejunal reconstruction: end-to-side or duct-to-mucosa pancreaticojejunal. If the diameter of the duct of Wirsung is larger than 5 mm, duct-to-mucosa pancreaticojejunal is easier to execute. In our series, all patients had an undilated duct of Wirsung, which is difficult to identify. Therefore, end-to-side pancreaticojejunal seems to be easier and faster. A comparison between duct-to-mucosa and end-to-side pancreaticojejunal reconstruction after pancreaticoduodenectomy revealed no significant differences in the rate of complications [20]. In our study, an end-to-side pancreaticojejunal was used to accomplish the pancreaticojejunal reconstruction and we had a pancreatic fistula rate of 20%. A recent comparative study has shown that division of the pancreatic parenchyma with vascular cartridges resulted in a significantly lower fistula rate compared with standard cartridges [21]. It is still unclear if the use of staple-line reinforcement reduces the risk of a pancreatic fistula [22]. The limitations of this study were its retrospective design and low number of patients. These problems can be overcome only by a large, prospective randomized trial, which would be difficult to accomplish owing to the infrequent diagnosis of patients with SPT of the pancreas.

## Conclusions

In conclusion, LCP is a safe and effective technique for resecting SPT of the neck and proximal body of the pancreas while preserving pancreatic endocrine and exocrine function, and the spleen. A minimally invasive approach ensures adequate treatment despite requiring the expertise of highly skilled laparoscopic surgeons.
